# Autophagy-related proteins are functionally active in human spermatozoa and may be involved in the regulation of cell survival and motility

**DOI:** 10.1038/srep33647

**Published:** 2016-09-16

**Authors:** I. M. Aparicio, J. Espino, I. Bejarano, A. Gallardo-Soler, M. L. Campo, G. M. Salido, J. A. Pariente, F. J. Peña, J. A. Tapia

**Affiliations:** 1 Cell Physiology Research Group, Department of Physiology, University of Extremadura, Caceres, Spain; 2 Neuroimmunophysiology and Chrononutrition Research Group, Department of Physiology, University of Extremadura, Badajoz, Spain; 3 Department of Biochemistry and Molecular Biology, University of Extremadura, Caceres, Spain; 4 Laboratory of Spermatology, Department of Animal Medicine, University of Extremadura, Caceres, Spain

## Abstract

Macroautophagy (hereafter autophagy) is an evolutionarily highly conserved cellular process that participates in the maintenance of intracellular homeostasis through the degradation of most long-lived proteins and entire organelles. Autophagy participates in some reproductive events; however, there are not reports regarding the role of autophagy in the regulation of sperm physiology. Hence, the aim of this study was to investigate whether autophagy-related proteins are present and functionally active in human spermatozoa. Proteins related to autophagy/mitophagy process (LC3, Atg5, Atg16, Beclin 1, p62, m-TOR, AMPKα 1/2, and PINK1) were present in human spermatozoa. LC3 colocalized with p62 in the middle piece of the spermatozoa. Autophagy activation induced a significant increase in motility and a decrease in PINK1, TOM20 expression and caspase 3/7 activation. In contrast, autophagy inhibition resulted in decreased motility, viability, ATP and intracellular calcium concentration whereas PINK1, TOM20 expression, AMPK phosphorylation and caspase 3/7 activation were significantly increased. In conclusion our results show that autophagy related proteins and upstream regulators are present and functional in human spermatozoa. Modification of mitochondrial proteins expression after autophagy activation/inhibition may be indicating that a specialized form of autophagy named mitophagy may be regulating sperm function such as motility and viability and may be cooperating with apoptosis.

Macroautophagy (hereafter autophagy) is an evolutionarily highly conserved cellular process among eukaryotes that participates in the maintenance of intracellular homeostasis through the degradation of most long-lived proteins and entire organelles. When autophagy is activated, a membrane cisterna called phagophore encloses a portion of cytoplasm, resulting in the formation of the autophagosome. Further, the outer membrane of the autophagosome fuses with the membrane of a lysosome, resulting in the degradative structure termed the autolysosome or autophagolysosome, where hydrolytic enzymes, supplied by the lysosome, degrade the cytoplasm-derived materials together with the inner membrane of the autophagosome^1^. Products resulted from the degradation are released back into the cytosol in order to recycle the macromolecular constituents and to generate energy to maintain cell viability. Autophagy can be selective and non-selective depending on the cellular component degraded. Nonselective autophagy is used for the turnover of bulk cytoplasm, whereas selective autophagy specifically targets damaged or superfluous organelles, and accordingly is identified with a unique name: mitophagy for selective mitochondria degradation by autophagy, pexophagy for peroxisomes, lysophagy for lysosomes, etc refs 2, 3, 4.

Phagophore, autophagosome and autophagolysosome formation is finely regulated by at least 30 autophagy-related proteins (Atg). Atg1 and Beclin 1 (mammalian homolog of Atg 6) participate in the early stages of this process. Further, protein associations among Atg5, Atg12, Atg16 and lipidation of Atg8 induce the autophagosome formation.

One of the mammalian homolog of yeast Atg8 is the Microtubule-associated protein light chain 3 (LC3) and exists in two forms, LC3-I and LC3-II. LC3-I is an 18-kDa polypeptide normally found in the cytosol, whereas the product of its proteolytic maturation (LC3-II, 16 kDa) resides in the autophagosomal membranes^5^,^6^. LC3-II has been widely used to study autophagy and it has been considered as an autophagosomal marker in mammals^7^.

Among the numerous proteins involved in the regulation of autophagy, mTOR (mammalian target of rapamycin) is a key component^8^. Under normal conditions, mTOR is inhibiting autophagy. Starvation conditions and environmental stress inactivate mTOR which results in an activation of autophagy. Other important regulators of autophagy include class I and class III PI3Ks and AMPK^9^,^10^.

With these regulators it is not a surprise that autophagy is physiologically activated under starvation and stressful conditions, and that its activation contributes to maintain cellular homeostasis providing an energy source when is demanded by the cell. However, by using chemical drugs or through regulation of essential genes, autophagy has been also involved in a wide spectrum of pathophysiological processes, such as: myopathies, neurodegenerative disorders and cancer^11^. In animal reproduction, autophagy activation in the oocyte participates in the elimination of sperm mitochondrial DNA (mtDNA) to prevent both the transmission of paternal mtDNA to the offspring and the establishment of heteroplasmy^12^,^13^,^14^,^15^; although this role is not completely clear, existing discrepancies among studies^16^,^17^,^18^. Autophagy markers, such as LC3-II and autophagosomes, have been previously detected in rat and mice spermatogenic cells^19^,^20^. In both studies, autophagy, measured as ratio of LC3-II/LC3-I, increased significantly after 12 h of culture^19^ and after testes heat treatment^20^. Interestingly, new reports have shown a critical function for Atg7 in the process of acrosome biogenesis, supporting the autolysosome origination hypothesis for acrosome formation in mice and it is also required for spermatozoa flagella biogenesis and cytoplasm removal during spermiogenesis^21^,^22^. Finally, LC3-II/LC3-I ratio has been related with sperm survival in equine spermatozoa^23^,^24^,^25^.

Although the primordial role of autophagy is to confer cell protection, it also has been shown to facilitate, antagonize or cooperate with apoptosis, serving either a pro-survival or pro-death function^26^. Ejaculated spermatozoa have been shown to display morphological and biochemical features that are typical of an apoptotic phenotype in somatic cells^27^,^28^,^29^,^30^,^31^,^32^ and, during spermatogenesis in the testis, apoptosis is required to accomplish an equilibrated ratio between somatic and germinal component^11^,^33^,^34^,^35^,^36^. Recently, it has been shown that autophagy and apoptosis cooperate to induce germ cell death during spermatogenesis, in mice^20^.

Despite the great importance that autophagy process has in the regulation of cell survival, to our knowledge, it has not yet been investigated in ejaculated spermatozoa of any specie. Hence, we aimed to investigate whether the proteins involved in the regulation of autophagy and in LC3-II processing are present in human spermatozoa. Also, we studied whether autophagy might be functional in human spermatozoa participating in the regulation of motility and viability. Finally, we questioned whether a possible link between autophagy and proteins related to apoptosis might exist in the regulation of human sperm survival.

## Results

### Identification of autophagy-related proteins in human spermatozoa

**Figure 1:**
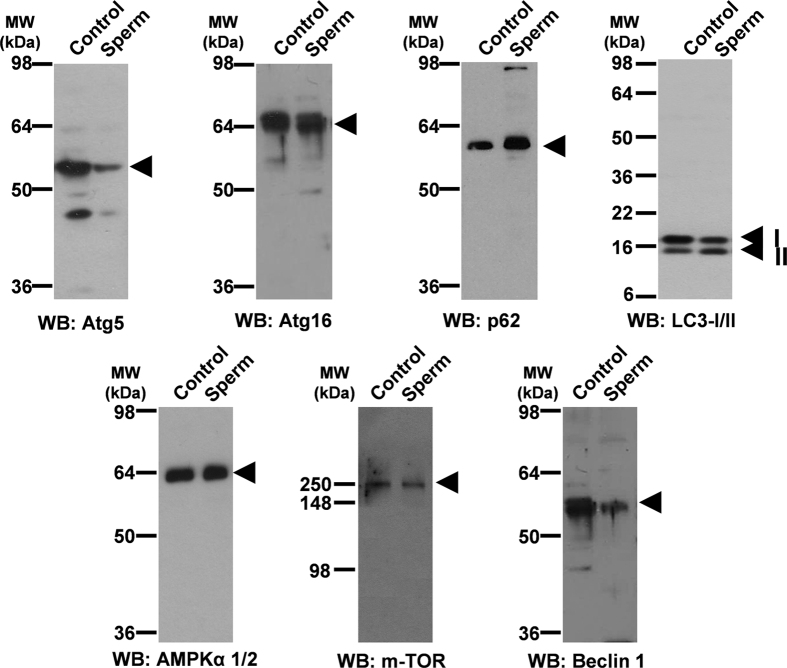
Identification of proteins related to autophagy pathway. 15 μg of proteins from whole cells lysates were loaded and separated by SDS-PAGE. Protein from rat brain was used as positive control for all proteins tested except for LC3 where the positive control was 293T cells with overexpressed human LC3 (10 μl). Immunoblotting was performed, as described in material and methods, with specific antibodies against: Atg 5, Atg 16, p62, LC3 (which recognizes both LC3-I and LC3-II), AMPKα1/2, m-TOR and Beclin 1 proteins.

Antibodies against proteins that participate in the autophagosome formation (Atg 5, Atg 16, p62 and LC3-II/I) and in autophagy regulation (AMPK, m-TOR and Beclin 1) were used. To accurately evaluate the immunoblotting results, human sperm lysates were used in parallel with a number of positive controls where the presence of these proteins has been previously described. Moreover, these controls allowed us to evaluate whether antibodies were useful to recognize the target proteins (Fig. 1).

All antibodies used unequivocally detected proteins corresponding to the molecular weight expected for the protein under study in both positive controls and in sperm samples (Fig. 1), indicating that human sperm contain the proteins necessary for the autophagy process.

### Variability of LC3-II/LC3-I ratio in fresh samples from different individuals

**Figure 2:**
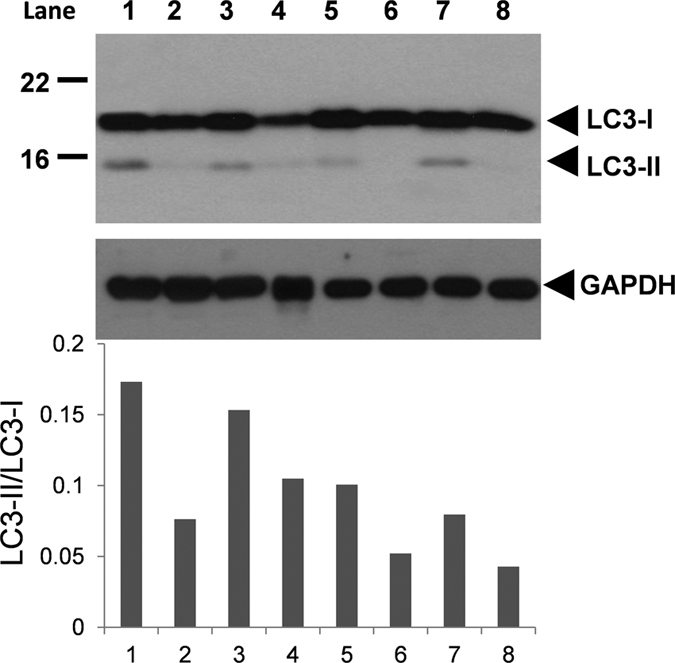
LC3-II/LC3-I ratio in basal conditions. Fresh semen was washed and proteins were extracted and further resolved in SDS-PAGE (see material and methods section). Immunoblotting was performed with an antibody that recognized both LC3-I and LC3-II proteins. Immunoblotting is a representative image of the protein expression of LC3 from 8 semen donors (lanes numbered from 1 to 8) from a total of 33. LC3-I and LC3-II were measured and autophagy flux was expressed as ratio of LC3-II/LC3-I.

Sperm proteins from 33 healthy donors were loaded and separated by SDS-PAGE, and immunoblotting was performed with an antibody that simultaneously recognizes both LC3-I and LC3-II. Figure 2 shows a representative image of 8 different individuals. Results indicate that autophagy, measured as a ratio of LC3-II/LC3-I, is active in basal conditions and highly variable among donors in fresh samples (Fig. 2). While fresh semen from donors 6 and 8 displayed the lowest ratio of LC3-II/LC3-I, donors 1 and 3 exhibited the highest ratio of LC3-II/LC3-I.

Having into account the great variability found among individuals, we assess whether the level of autophagy could be related to semen quality parameters. Table 1 shows that level of autophagy was highly and negatively correlated with concentration of spermatozoa per ml (p = 0.016), concentration of spermatozoa in the ejaculate (p = 0.023) and progressive motility (p = 0.042), and positively correlated with viability (p = 0.032).

### Expression of LC3-I and LC3-II after incubation

**Figure 3:**
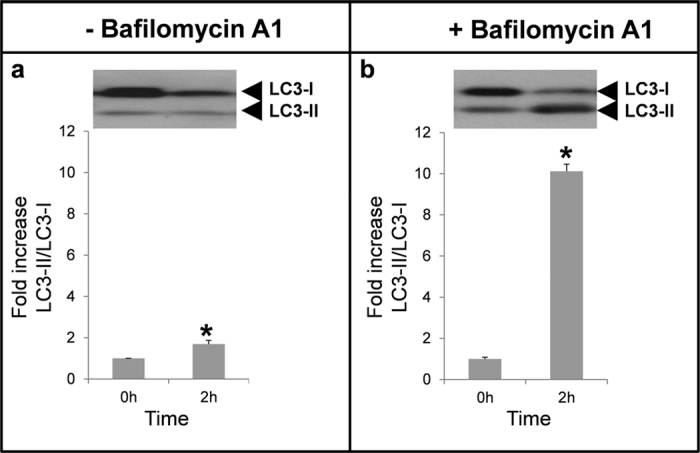
LC3-II/LC3-I ratio in incubated samples. Spermatozoa were incubated for 2 hours in absence (**a**) or presence (**b**) of bafilomycin A1 (100 nM) at 37 °C. Proteins were extracted, loaded and separated by SDS-PAGE. LC3 was detected by immunoblotting (see material and methods section). Results are expressed as increase of LC3-II/LC3-I ratio respect to fresh samples (T0). Columns with asterisk indicate significant differences (P < 0.05) respect to T0. n = 4.

To study whether autophagy is an active process in ejaculated sperm, we incubated the samples for 2 hours at 37 °C. After incubation, there was a considerable decrease in LC3-I while LC3-II intensity was not modified, compared with fresh samples (Fig. 3a). This could be the result of the LC3-I conversion to LC3-II and further degradation of LC3-II in the autophagosomes. To assess this hypothesis, spermatozoa were incubated in presence of bafilomycin 1, which impairs LC3-II degradation. In presence of bafilomycin 1, intensity of the protein band corresponding to LC3-II was remarkably higher (Fig. 3b) compared to LC3-II in samples after 2 hours of incubation without the drug (Fig. 3a) indicating that degradation of the LC3-II might occur in spermatozoa. Independent of the conditions, with or without bafilomycin A1, the ratio of LC3-II/LC3-I was significantly higher after 2 hours of incubation, compared to fresh samples, indicating that conversion of LC3-I to LC3-II is an active process in human spermatozoa.

**Figure 4:**
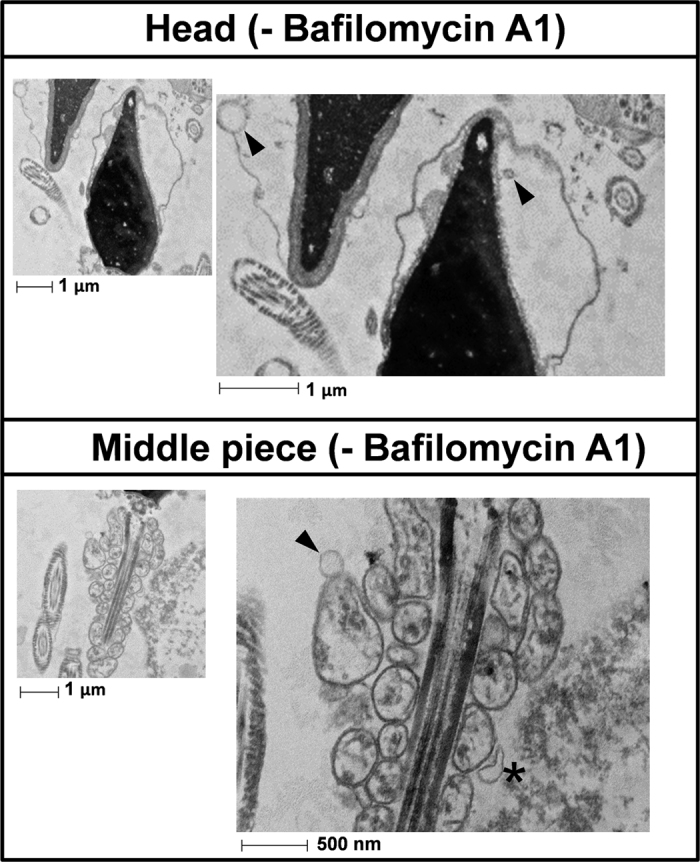
Transmission electron microscopy analysis without bafilomycin A1. Cells were prepared as described in the method section. Figure 4 shows representative transmission electron microscopy photographs of the head and the middle piece of the spermatozoa incubated for 2 hours in absence of bafilomycin A1. Vesicles are marked with arrows. Vesicle in the middle piece marked with an asterisk may represent the beginning of the process of sequestration. n = 3.

**Figure 5:**
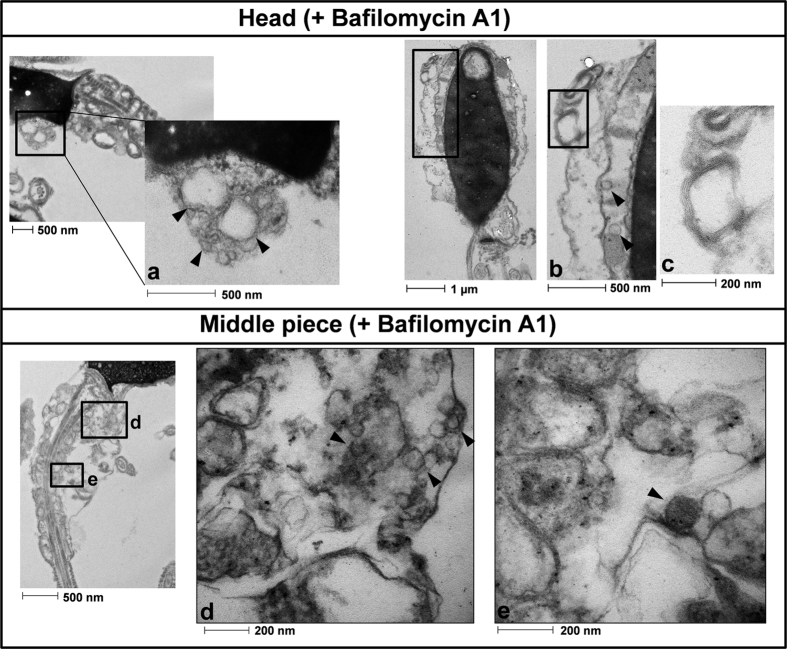
Transmission electron microscopy analysis with bafilomycin A1. Cells were prepared as described in the method section. Figure 5 shows representative transmission electron microscopy photographs from spermatozoa incubated for 2 hours in presence of bafilomycin A1. Vesicles found in the head (**a,b**) are marked with arrows. The image (**c**) shows a membrane lamella in the head of the spermatozoa which may correspond to the multilamellar bodies associated with the autophagy process. Images (**d,e**) correspond to photographs obtained from the middle piece of the spermatozoa. Some of the vesicles found are marked with an arrow n = 3.

Spermatozoa incubated for 2 hours in presence or absence of bafilomycin A1 were also analyzed by electron transmission microscopy (Figs 4 and 5). In absence of bafilomycin A1 (Fig. 4) we hardly observed vesicles either in the head or the middle piece of the tail. The vesicle observed in the middle piece, marked with an asterisk, may be displaying the first steps of the autophagy process, when the autophagosome begins to form (in this case around a mitochondrion) as a structure called phagophore assembly site (or PAS). When the cells were incubated in presence of bafilomycin A1 we could observe a high amount of vesicles in the head and middle piece of the spermatozoa (Fig. 5a,b,d,e respectively), which might correspond to autophagy vesicles. In addition, in the head we observed a structure which might correspond to those structures related to the autophagic process called multilamellar bodies (Fig. 5c). In the middle piece of the same cell, immature autophagic vacuoles characterized by an electron density equivalent to cytoplasm coexist with late vesicles, in which catabolic processes have been already started (characterized by an increased electron density) (Fig. 5d,e).

### LC3 immunolocalization

**Figure 6:**
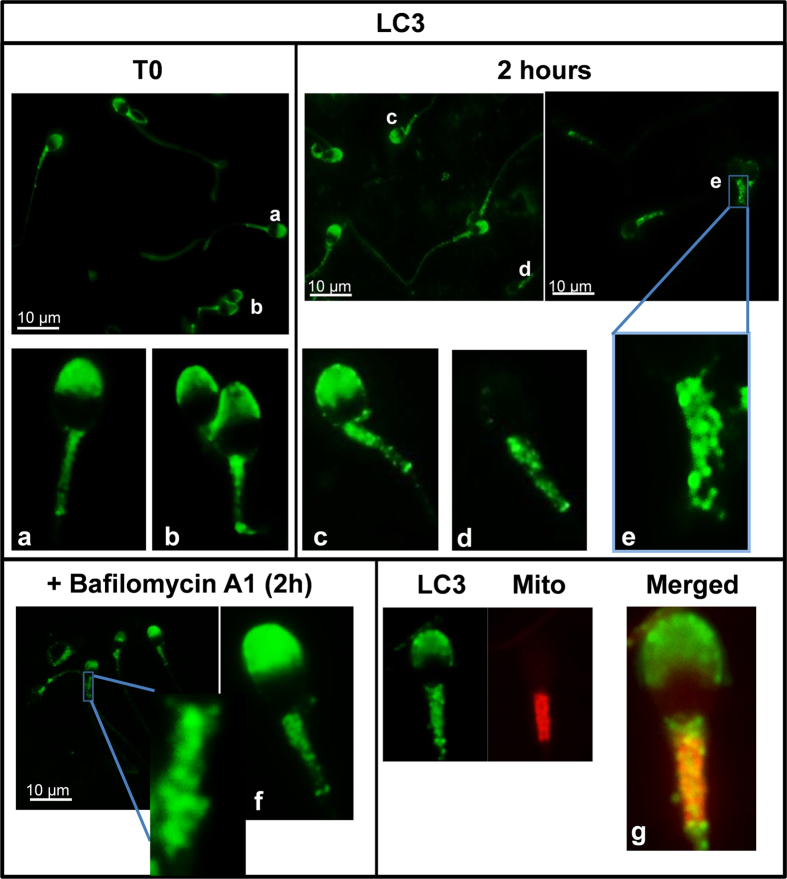
LC3-immunolocalization in fresh and incubated sperm cells. Localization of LC3 in sperm cells was performed as described in materials and methods section (indirect immunofluorescence) with anti-LC3 antibody (1/250). LC3 protein was visualized in green. Figures (**a**,**b)** (spermatozoa from fresh samples) (**c–e)** (spermatozoa after 2 hours of incubation) (**f**) (spermatozoa after 2 hours of incubation with bafilomycin A1) are representative areas digitally augmented showing the localization of LC3. Figure (**g**) shows a representative area digitally augmented from merged images of LC3 and cells stained with MitoTracker Red CMXRos. Places where both LC3 and mitochondria colocalize are observed in yellow.

Immunofluorescence analysis revealed that, in fresh samples, LC3 was homogenously localized in middle piece of the tail in all cells. Additionally most of the cells appeared with the acrosome region stained (72%) (Fig. 6a,b). In the sperm head LC3 showed two patterns of distribution: signal was uniformly distributed in the acrosomic region (Fig. 6a) or more concentrated in the apical region of the acrosome (Fig. 6b). After 2 hours of incubation only 40% of the cells had the acrosome region stained (Fig. 6c). In contrast to those observed in fresh samples where the signal in the middle piece of the tail was diffuse, after 2 hours of incubation the middle piece of the spermatozoa exhibited an evident punctate fluorescence pattern (Fig. 6e). Same pattern was observed after treatment with bafilomycin A1 (Fig. 6f), although in this case the percentage of cells with the acrosome stained was higher (61%) than in the cells incubated for 2 hours without bafilomycin A1. Finally, to assess whether LC3 colocalized with mitochondria, sperm cells were stained simultaneously with MitoTracker Red CMXRos and LC3 labeled with alexa 488. Results showed colocalization between LC3 and mitochondria (Fig. 6g) in the middle piece but not in the acrosome, as expected.

**Figure 7:**
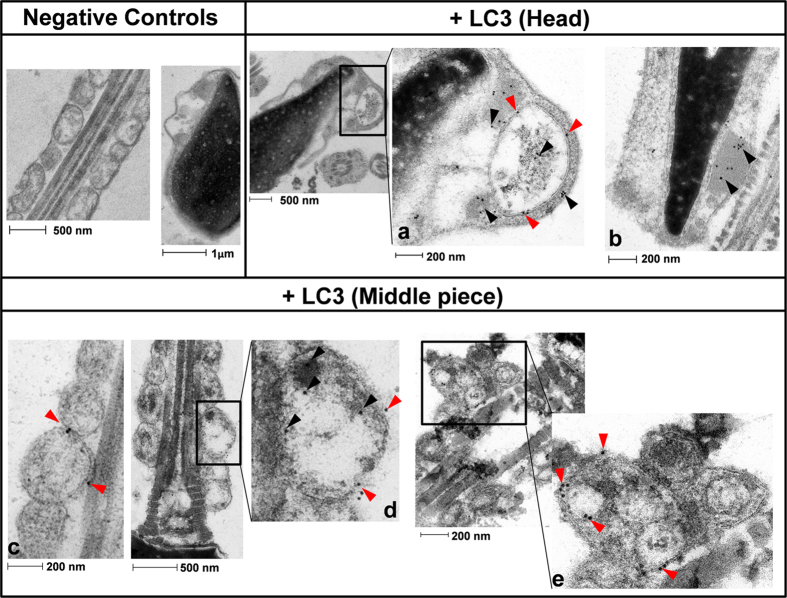
LC3 immunogold labeling. Spermatozoa were incubated in presence of bafilomycin A1 for 2 hours. Then, cells were prepared as described in material and methods. Negative controls are representative images from the head and the middle piece where primary Ab was omitted. Figures (**a,b**) are representative areas of the head. Figures (**c**,**d**,**e**) are digitally augmented images from mitochondria of the middle piece of the spermatozoa. Some of the LC3 proteins labeled with immunogold particles are marked with arrows; red arrows indicate gold particles (LC3) located on the membranes.

The distribution of LC3, labeled with immunogold particles, was also studied by electron transmission microscopy. The head of the spermatozoa (Fig. 7a,b) often showed vesicles with double-layered membrane, more than likely containing cellular debris. LC3 (gold particles) was mainly localized to the membrane and inside the vesicles, which may indicate the presence of autophagic vesicles in the head of the cells (Fig. 7a). In addition, immunogold-labeled LC3 was also frequently located over electron-dense structures in the head (Fig. 7b). In the middle piece of the spermatozoa, gold particles are prevalently present around the mitochondria, likely attached to the mitochondrial membrane itself (Fig. 7c) or to membranous and non-membranous structures over the surface of these organelles (Fig. 7d). In addition, Fig. 7e displays a less common finding, consisting in double-layered membrane vesicles containing smaller membranous structures and debris, which likely correspond to autophagic vesicles. Inside and around these vesicles gold particles are mostly associated to the membranes. Finally, negative controls of sperm incubated without the primary Ab (Fig. 7a) showed no immunogold labeling in the corresponding regions.

### LC3 colocalization with p62

p62 or sequestosome 1 (SQSTM1) is localized to the autophagosome via LC3 II-interaction and it is further degraded into these structures^37^.

**Figure 8:**
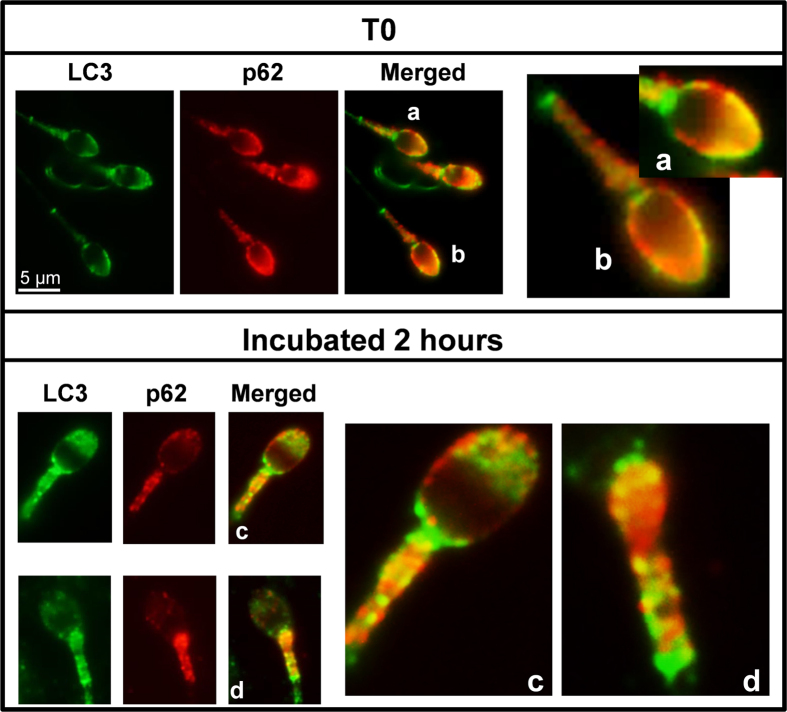
Colocalization of LC3 with p62. Colocalization of LC3 with p62/SQSTM1 sperm cells was performed as described in materials and methods section (indirect immunofluorescence) with specific antibodies: Anti-LC3 (1/250) and anti-p62 (1/200). LC3 protein was visualized in green and p62 in red. The figures (**a**,**b**) (spermatozoa from fresh samples) and (**c,d**) (spermatozoa after 2 hours of incubation) are representative areas digitally augmented showing the colocalization of LC3 and p62.

We performed colocalization studies between p62 and LC3. p62 was localized in the acrosome region and in the middle piece of the spermatozoa (Fig. 8a–d). In the middle piece p62 exhibited punctate structures. Immunofluorescence experiments with antibodies against p62 and LC3 in fixed cells revealed that p62 colocated with LC3 in the apical region of the acrosome at time 0 (without incubation) (Fig. 8a,b). After 2 hours of incubation, colocalization of both proteins was mainly detected in the middle piece of the tail (Fig. 8c,d).

### Effect of autophagy modulation on sperm viability and motility

Once we have observed that conversion of LC3-I to LC3-II is an active process in human spermatozoa, we aimed to investigate whether the modulation of this pathway might participate in the regulation of human sperm physiology. For this purpose, we used two drugs widely used in the inhibition and activation of autophagy in somatic cells: chloroquine and rapamycin.

**Figure 9:**
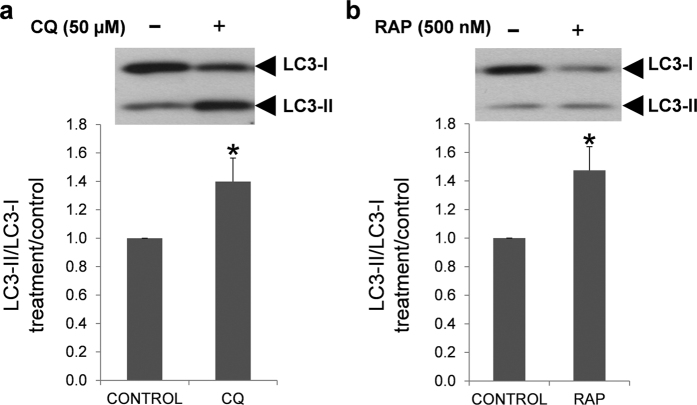
Effect of chloroquine and rapamycin on LC3-I and LC3-II expression. Human spermatozoa were incubated for 2 hours at 37 °C in presence or absence of chloroquine (**a**) and rapamycin (**b**). Proteins were extracted and resolved by SDS_PAGE. Immunoblotting was performed with anti-LC3 antibody (described in materials and methods section). Results are expressed as increase of LC3-II/LC3-I ratio respect to control samples (containing only vehicle). Columns with asterisk indicate significant differences (P < 0.05) respect to control. n = 4. CQ: chloroquine; RAP: rapamycin.

First of all, we investigated whether these drugs were also able to modulate the conversion of LC3-I to LC3-II in human spermatozoa. Treatment with chloroquine increased significantly LC3-II, and hence increased LC3-II/LC3-I ratio (Fig. 9a). LC3-II/LC3-I ratio was also increased after incubation with rapamycin (Fig. 9b).

**Figure 10:**
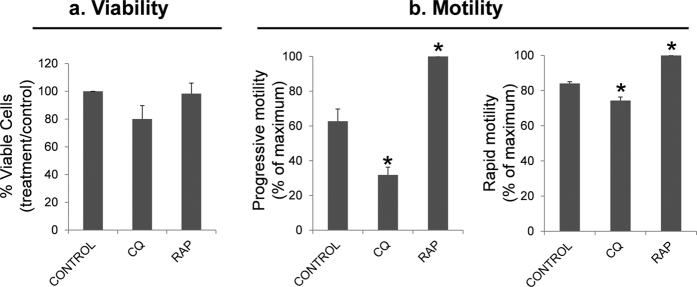
Effect of chloroquine and rapamycin on spermatozoa viability and motility. Sperm cells were incubated in presence of chloroquine (50 μM) and rapamycin (500 nM) for 2 hours at 37 °C. Further, cells were incubated with SYBR 14, propidium iodide (PI) and Hoechst 33342 and examined by flow cytometry (described in material and methods). Sperm motility was assessed by CASA. (**a**) graphic shows the percentage of SYBR 14 positive and PI negative cells and results are expressed as the increase respect to control ± SEM (containing vehicle) (n = 6); (**b**) figures represent the percentage of spermatozoa with progressive and rapid motility. Results are represented as percentage of maximum ± SEM (n = 4). Columns with asterisk indicate significant differences (P < 0.05). CQ: chloroquine; RAP: rapamycin.

Once tested that both chloroquine and rapamycin modulated the LC3 conversion, we assessed their effects in viability. Neither chloroquine nor rapamycin had significant effect on viability, however chloroquine tended to decrease the percentage of viable (20% less than control) being almost statistically significant (p = 0.057) (Fig. 10a).

Incubation of human spermatozoa with chloroquine for 2 hours at 37 °C decreased significantly the percentage of spermatozoa with progressive and rapid motility (Fig. 10b). Also, some velocity parameters, such as VSL, STR and WOB decreased after treatment with this autophagy inhibitor (Table 2). In contrast to chloroquine, treatment with rapamycin had a positive effect on motility increasing progressive motility and the percentage of rapid spermatozoa (Fig. 10b), while velocity parameters were not significantly modified (Table 2).

### Intracellular pH, ATP and calcium concentration

**Figure 11:**
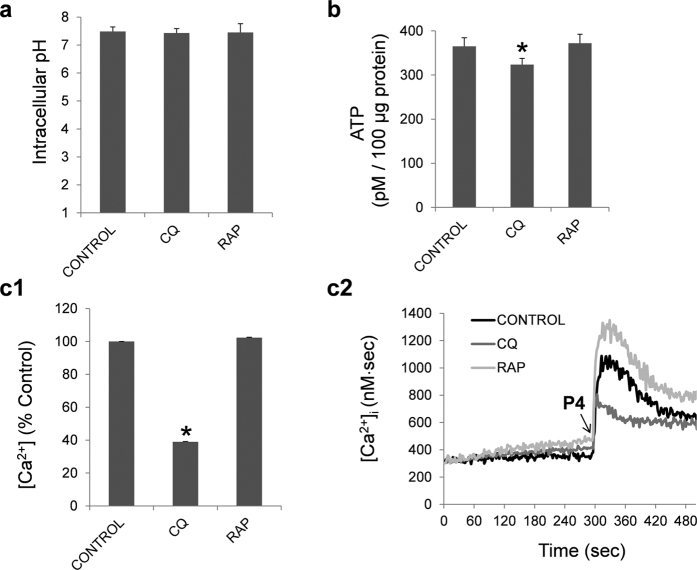
Intracellular pH, ATP and calcium concentration. Intracellular pH (**a**), ATP concentration (**b**) and intracellular calcium concentration (**c1,c2**) were studied in human spermatozoa, as described in material and methods section, after 2 hours of incubation with chloroquine (50 μM) and rapamycin (100 nM). ATP results are expressed as pM concentration of ATP in 100 μg of protein in each sample. Fluorescence of FURA-2-AM was recorded and changes in intracellular [Ca^2+^]i were monitored every second (**c1**). Area under the curve after progesterone addition was calculated and expressed as percentage of treatment vs control (**c2**). Results are expressed as mean ± SEM from 4 independent experiments. Columns marked with *indicate significant differences compared to control (containing vehicle) (P < 0.05). CQ: chloroquine; RAP: rapamycin.

Incubation of human spermatozoa with chloroquine and rapamycin for 2 hours at 37 °C did not modify the pH intracellular (Fig. 11a), whereas ATP concentration decreased significantly after 2 hours of treatment with chloroquine (Fig. 11b).

Next, we aimed to study the effect of chloroquine and rapamycin in intracellular calcium concentration. For this purpose, we used the agonist progesterone which induces an increase in the cytosolic free calcium concentration in human spermatozoa^38^. The response of the chloroquine treated cells to progesterone was significantly lower (Fig. 11c1) resulting in low intracellular calcium concentration (Fig. 11c2). Treatment with rapamycin had no significant effect on the intracellular calcium concentration (Fig. 11c1,c2), compared to control (containing vehicle).

### Effect of chloroquine and rapamycin in AMPKα 1/2 phosphorylation (Thr 172)

**Figure 12:**
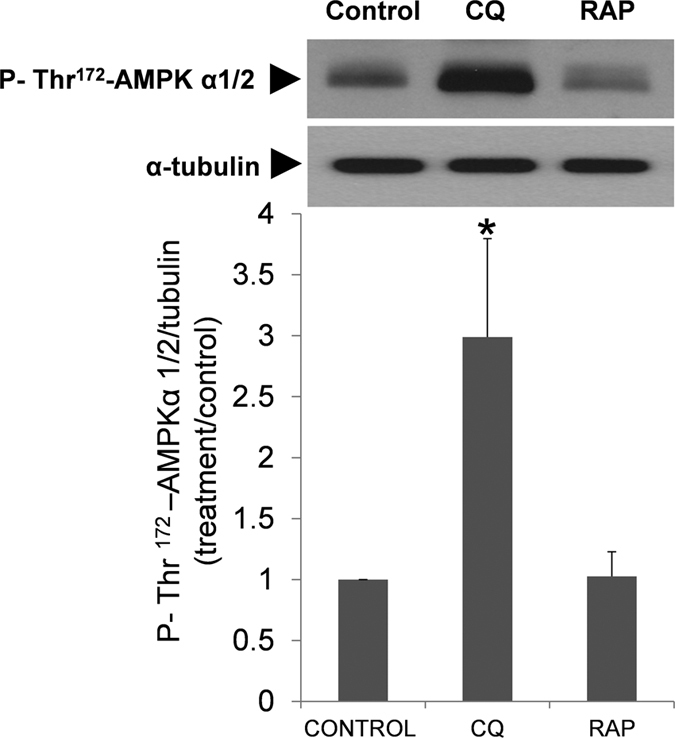
Regulation of AMPKα 1/2 phosphorylation by chloroquine and rapamycin. Spermatozoa were incubated in presence of chloroquine (50 μM) and rapamycin (100 nM). Proteins were then extracted and analyzed by immunoblotting. AMPKα 1/2 phosphorylation was studied with a phosphospecific antibody that recognized phosphorylation on Thr 172. These membranes were also incubated with tubulin for normalization. Results represent the fold-increase of P-Thr^172^-AMPKα 1/2 normalized with tubulin. Columns with asterisk indicate significant differences (P < 0.05) respect to control (vehicle). n = 4. CQ: chloroquine; RAP: rapamycin.

AMPK activation is regulated by ATP content, being considered as a sensor of the energetic status in the cells^39^. Human spermatozoa where incubated in presence of chloroquine and rapamycin for 2 hours at 37 °C (Fig. 12). Chloroquine induced a significant increase in the phosphorylation (Thr 172) of AMPKα 1/2, thus increasing AMPK activity. Rapamycin, as expected, had no effect in the phosphorylation of this protein as its target (m-TOR) is downstream to AMPK.

### Quantification of PINK1 and TOM20

Colocalization of LC3 with p62 and mitochondria in the middle piece of the tail, together with a decrease in motility, ATP and calcium when autophagy is inhibited may be related to modifications in the mitochondria network. Hence, we aimed: a) to study PINK1 protein, which has been noted as a sensor of the mitochondrial status and is responsible for the degradation of mitochondria through autophagy (mitophagy)^40^; b) to investigate TOM20 protein which is an outer mitochondrial membrane protein widely used for monitoring mitochondrial mass during mitophagy^41^,^42^; c) to determine the mitochondrial population levels by flow cytometry with MitoTracker Deep Red (MTDR) which is used to analyze the mitophagy process^43^.

**Figure 13:**
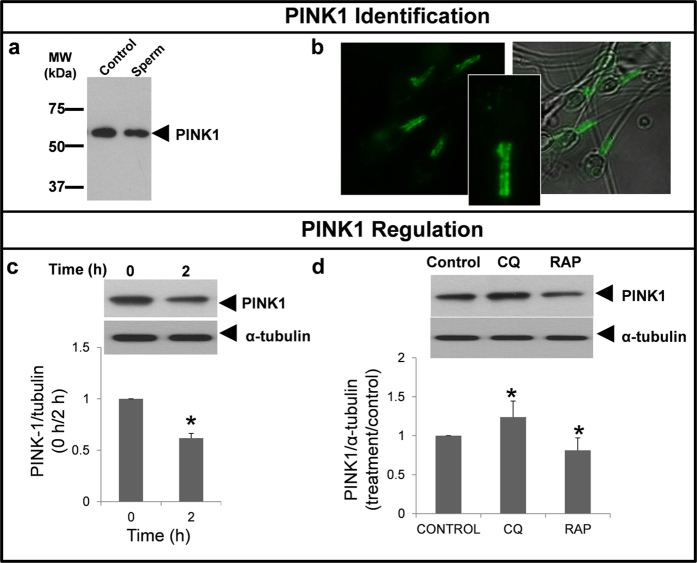
PINK1 regulation by chloroquine and rapamycin. Identification of PINK1 (**a**) whole lysate from rat brain cells was used as positive control for protein identification. 15 μg of proteins from rat brain and human spermatozoa were loaded, separated by SDS-PAGE and immunoblotting was performed with a specific antibody against PINK1. PINK1, green signal, was studied by immunofluorescence, as described in material and methods in fresh samples (**b**). Regulation of PINK1 protein was studied in fresh and after 2 hours of incubation (**c**) and in the presence of chloroquine (50 μM) and rapamycin (100 nM) (**d**). Proteins were extracted, resolved and detected with specific antibody. Data from PINK1 was normalized respect to α-tubulin. Columns with asterisk indicate significant differences (P < 0.05) respect to control (containing vehicle). n = 4. CQ: chloroquine; RAP: rapamycin.

**Figure 14:**
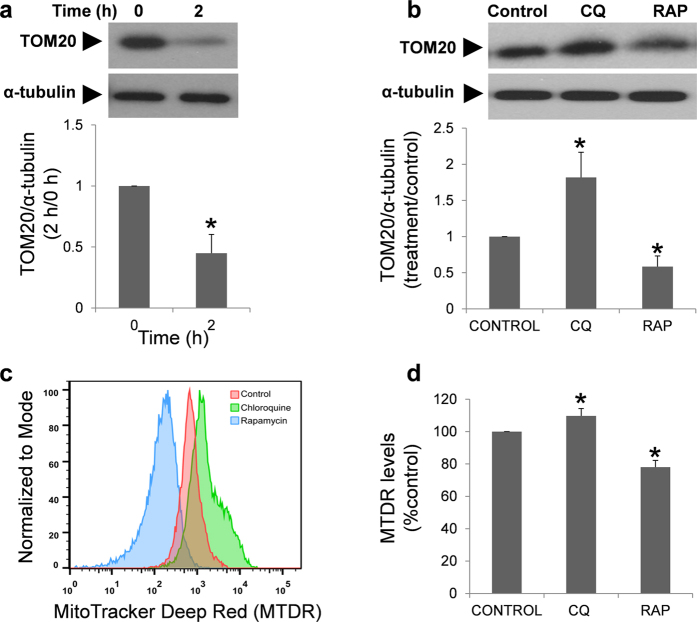
TOM20 protein and MitoTacker Deep Red (MTDR) fluorescence regulated by chloroquine and rapamycin. Regulation of TOM20 protein was studied in fresh and after 2 hours of incubation (**a**) and in the presence of chloroquine (50 μM) and rapamycin (100 nM) (**b**). Proteins were extracted, resolved and detected with specific antibody. Data from TOM20 was normalized respect to α-tubulin. Columns with asterisk indicate significant differences (P < 0.05) respect to control (containing vehicle). n = 4. CQ: chloroquine; RAP: rapamycin. MTDR (**c**,**d**) was used to determine mitochondrial staining by flow cytometry in cells incubated in the presence of chloroquine (50 μM) and rapamycin (100 nM). Figure (**c**) is a representative and normalized histogram of the MTDR fluorescence obtained after treatment with rapamycin (blue) and chloroquine (green) for 2 hours. Figure (**d**) represents the mean of MTDR fluorescence of four different experiments and normalized respect to control. Columns with asterisk indicate significant differences (P < 0.05) respect to control (containing vehicle). CQ: chloroquine; RAP: rapamycin.

PINK1 antibody recognized one protein band corresponding to the expected molecular weight (∼60 kDa) either in the positive control and human sperm lysates (Fig. 13a). Immunofluorescence study revealed that PINK1 is specifically located in the middle piece of the tail in human spermatozoa (Fig. 13b). After two hours of incubation the quantity of PINK1 (Fig. 13c) and TOM20 (Fig. 14a) decreased significantly. Incubation with chloroquine induced a significant increase in PINK1 (Fig. 13d), TOM20 (Fig. 14b) and MTDR fluorescence (Fig. 14d), while rapamycin clearly decreased the quantity of both proteins and MTRD fluorescence in human spermatozoa compared to control (Figs 13d and 14b,d respectively).

### Caspase 3/7 activation

As autophagy and apoptosis can be linked and can cooperate to regulate cell survival in somatic cells, our final objective was to investigate caspase 3/7 activation upon treatment with chloroquine and rapamycin.

**Figure 15:**
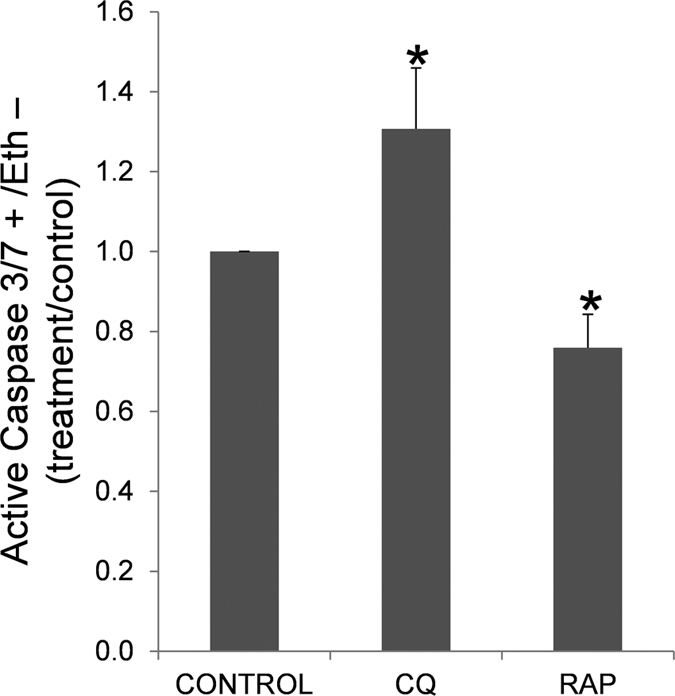
Caspase 3/7 activation. Sperm cells were incubated in presence of chloroquine (50 μM) and rapamycin (500 nM) for 2 hours at 37 °C. After the incubation sperm cells were incubated with CellEvent Caspase-3/7 Green Detection Reagent, ethidium homodimer (Eth) and Hoechst 33342 and examined by flow cytometry as described in material and methods. Graphic shows the percentage of Caspase 3/7 positive cells and Eth negative cells (dead cells), expressed as increase of active caspase 3/7 in treatments respect to control (containing vehicle). Columns with asterisk indicate significant differences (P < 0.05) respect to control (vehicle). n = 6. CQ: chloroquine; RAP: rapamycin.

Chloroquine induced a significant increase in the activation of caspases 3/7, while rapamycin statistically decreased the activation of these caspases, compared to control samples (Fig. 15).

## Discussion

Autophagy is a very intriguing pathway controlled by multiple proteins: those involved in the regulation of the formation of the autophagolysosome (at least 30 autophagy-related proteins (Atg) and those proteins involved in the activation of the process such as Beclin 1, PI3K class III, m-TOR, AMPK, among others^1^,^11^. In the present study we show that human spermatozoa contain both series of proteins: Atg 5, Atg 16, p62 and LC3 involved in the formation of the autophagolysosome and m-TOR, Beclin 1 and AMPK described as the main regulators of autophagy^1^,^10^,^11^. Despite each protein is important in the regulation of this pathway, the most used for monitoring the flux of autophagy is the ratio of LC3-II/LC3-I^5^. LC3-I is localized in the cytosol and, after autophagy activation, is conjugated to phosphatidylethanolamine to form LC3-phosphatidylethanolamine conjugate (LC3-II), which is recruited to autophagosomal membranes for being further degraded with all the autophagolysosome content^5^. The incubation of spermatozoa for 2 hours at 37 °C induced a significant increase in the ratio of LC3-II/LC3-I. This increase was higher with the addition of bafilomycin A1, which blocks the fusion of autophagosomes with lysosomes, leading to an accumulation of autophagosomal structures and hence inhibiting the degradation of LC3-II^44^. These results indicate that conversion of LC3-I to LC3-II is taking place in human spermatozoa and that LC3-II is subsequently degraded. Under transmission electron microscopy (TEM) and in presence of bafilomycin A1, large amount of vesicles (electron-permeable and non-permeable vesicles) were found in the head and middle piece of the sperm, which might correspond to autophagosomes or autophagolysosomes^45^,^46^. In the head of the spermatozoa we also found structures similar to multilamellar bodies (MLBs), which are formed when selective resistance to lysosomal degradation within the autophagic vacuole occurs^45^,^47^. In the absence of bafilomycin A1 vesicles were hardly found. These results might indicate that autophagosome formation and autophagolysosome degradation in human spermatozoa require less than 2 hours, as it occurs in somatic cells, where most autophagosomes are very short-lived, with a half-life of approximately 20 minutes^48^,^49^.

In order to study the nature of these vesicles bafilomycin-treated human sperm samples were analyzed by TEM after the immunogold labeling of LC3. Under such experimental conditions we did not longer observe such amount of vesicles found previously in the spermatozoa, which may be due to a change in the fixative protocol in order to improve the antigen-antibody binding. However, after the immunogold labeling of LC3 we detected in the head of the spermatozoa double-layered membrane vesicles containing cellular debris that consistently appeared marked with immunogold particles and would likely correspond with autophagic vesicles. In the middle piece of the bafilomycin-treated human spermatozoa the gold particles were often detected around mitochondria, but occasionally we also observed vesicles containing small membranous structures and debris that were profusely marked with immunogold particles. Again, these structures would probably correspond with autophagic vesicles. Immunogold particles were also often detected in locations unrelated to membranes. It is worth mentioning that those gold particles which appear to be attached to the membranous structures, both in the head and in the middle piece, likely correspond to phosphatidylethanolamine-conjugated LC3, namely lipid-bound LC3 or LC3-II, while the remaining locations of the gold particles may correspond to the unprocessed, soluble form of LC3 or LC3-I. However, since the anti-LC3 antibodies used in this study recognize both LC3-I and LC3-II forms it is impossible to completely ensure such equivalence.

To further characterize the autophagy process in human spermatozoa we investigated p62, which may be also used as a marker to study the autophagic flux because p62 accumulates when autophagy is inhibited whereas is degraded when autophagy is induced^37^. Cell stress and infection promote the formation of ubiquitinated aggregates. These ubiquitinated structures can be recognized by the autophagy receptor, p62, and then targeted for autophagic degradation by interactions of p62 with both ubiquitin chains and LC3^50^,^51^. In fresh samples, LC3 and p62 colocalized in the acrosome region of the spermatozoa, while the main colocalization of both proteins was found in the middle piece of the sperm displaying the typical punctate staining observed after autophagy activation after 2 hours of incubation^5^.

Sperm is a compartmentalized cell which can be basically divided into two major morphologically distinct compartments: head and tail. Because both, head and tail, are so structurally distinct and physically different, it is plausible to think that autophagy may have unrelated functions in the sperm, such as the activation of flagellar motility or the acrosomal exocytosis. However, there are models favoring the concept that the flagellum senses external signals and communicates with the head by second messengers to affect sperm functions such as acrosomal exocytosis^52^. As the signal from the head disappear after 2 hours, it is also possible to think that LC3 and p62 translocate from the head to the tail to exert their functions in the middle piece.

The observed autophagic hallmarks in the middle piece of the spermatozoa, which is known to contain a large number of mitochondria, may be suggesting that a selective form of autophagy, termed mitophagy, may occur in the spermatozoa. Mitophagy is the responsible for the elimination of dysfunctional or damaged mitochondria contributing to mitochondrial quality control and, thereby, to the maintenance of efficient cellular metabolism^4^,^53^. An example of mitophagy is the probable elimination of the sperm-derived mitochondria in the fertilized oocyte, thereby allowing for exclusively maternal inheritance of mitochondrial DNA^12^,^15^,^54^. Mitophagy requires PINK1, a Ser/Thr kinase that contains a mitochondrial targeting sequence allowing for its mitochondrial localization. In healthy mitochondria, PINK1 is constitutively imported to the inner membrane where it is cleaved by several proteases and ultimately proteolytically degraded. Loss of mitochondrial membrane potential impedes import of PINK1 to the inner membrane, thereby stabilizing intact PINK1 on the mitochondrial outer membrane. In this manner, PINK1 accumulation serves as a sensor for mitochondrial damage. Accumulation of PINK1 on the mitochondrial surface induces translocation of Parkin from the cytosol to damaged mitochondria. Following the Parkin-mediated ubiquitination of proteins, these substrates are then bound by p62, a key factor in selective autophagy^55^, which then delivers mitochondria to the autophagosome via interaction with LC3^56^. Finally, damaged mitochondria are degraded by autophagy^40^,^53^. Apart from LC3 and p62, PINK1 protein was also detected in the middle piece of human spermatozoa, and after two hours of incubation it displays a punctate form (results not shown). When spermatozoa were incubated in presence of chloroquine there was an accumulation of this protein, which may be explained by the inhibitory effect that this compound has on autophagy/mitophagy^57^. In addition, the quantity of TOM20, an outer mitochondrial membrane protein widely used for monitoring mitochondrial mass during mitophagy^41^,^42^,^58^, increased significantly after treatment with chloroquine. In contrast to chloroquine, rapamycin (which activates autophagy through m-TOR inhibition) decreased the quantity of PINK1 and TOM20 proteins, more than likely indicating degradation of these proteins^59^. Recently it has been published a new method to assess mitophagy by flow cytometry which consist of the study of the changes in the fluorescence levels of MTDR^43^. In our conditions, chloroquine increased the fluorescence of MTDR whereas rapamycin decreased it, in concordance with previous results^43^. Therefore, immunolocalization of the vesicles and proteins in the middle piece of the spermatozoa (by immunofluorescence and by immunogold labeling) and the modifications in the expression of mitochondrial proteins and MTDR fluorescence after treatment with autophagy modulators are strongly suggesting that a mitophagy-like mechanism may occur in human spermatozoa.

Autophagy/mitophagy is regulated by AMPK, which is a conserved sensor of intracellular energy. In response to decreases in intracellular ATP, AMPK is activated (phosphorylated) and serves as a metabolic checkpoint, restoring ATP levels^39^. The increase in the phosphorylation of AMPK after incubation with chloroquine could be reflecting the decreased in ATP observed after treatment with this drug^39^.

Mitochondria localized in the middle piece of the spermatozoa are subcellular organelles crucial for the life of the cell. They are sensors of metabolic homeostasis, and regulate the levels of intracellular signaling molecules, such as calcium and ATP production. In human sperm cells, mitochondrial dysfunction has been associated with low motility, high ROS production, low motility and infertility (reviewed in ref. 60). Our next objective was to investigate whether the modulation of the LC3 conversion with chloroquine and rapamycin, two drugs widely used for monitoring autophagy in somatic cells^61^,^62^, may affect sperm physiology. The treatment with chloroquine, which inhibits autophagy, decreased viability, motility, ATP content, and calcium concentration. In agreement with our results, it has previously been demonstrated that chloroquine can inhibit motility and viability in human spermatozoa, although in these previous reports the effects of chloroquine never have been linked with autophagy^63^,^64^. Hargreaves C.A. *et al*. demonstrated that treatment with 100 μg/ml of chloroquine for 24 hours decreased motility and viability^63^. Differences in the concentration used (100 μg/ml vs. 25.8 μg/ml in our work) and/or the time of incubation (24 h vs. 2 h) could explain the absence of a significant effect in the percentage of viable cells after the treatment with chloroquine, although in our study chloroquine tended to decrease viability by almost 20%, compared to control. In contrast to chloroquine, rapamycin had a positive effect on motility without effect in viability, ATP content and intracellular calcium concentration. From our knowledge, results obtained with rapamycin cannot be compared with other studies as it has not been previously used in spermatozoa, although in somatic cells^60^ m-TOR signaling regulates mitochondrial turnover, decreasing ROS production and increasing mitochondrial respiration when is inhibited or mutated^65^, which could explain the increase observed in motility and the maintenance of viability in human spermatozoa. In addition, in somatic cells, when clearance of mitochondria is impaired there is an increase in ROS production, loss of homeostasis cellular, decreased mitochondrial membrane potential and ATP content^66^.

The mitochondrion has been noted as a switch between apoptosis and autophagy^67^. If autophagy is blocked and the damaged mitochondria are not removed, the mitochondria undergoing mitochondrial membrane potential loss can prime apoptosis^67^. Hence, we also aimed to study whether a relationship exists between both pathways. When autophagy/mitophagy in human spermatozoa was inhibited with chloroquine, the percentage of caspase 3/7 positive cells significantly increased, while autophagy/mitophagy activation with rapamycin caused a significant decrease in caspase 3/7, all of which is in agreement with previous results in somatic cells^68^,^69^. Although it is not clear whether apoptosis in an active process in human spermatozoa, morphological and biochemical features that are typical of this process have been correlated with infertility^30^. Caspase-3 activity is increased after disruption of mitochondrial membrane potential and its activation has been correlated with low motility in human spermatozoa^70^,^71^,^72^. Therefore, the results obtained are suggesting that autophagy/mitophagy could act as a pro-survival mechanism allowing the clearance of mitochondria and maintaining the homeostasis and metabolism in the spermatozoon. However, when degradation is inhibited, accumulation of damaged organelles might cause detrimental effects in the cell inducing the activation of caspases 3/7. In mice germ cells, autophagy and apoptosis are also linked but, in contrast to our results, both processes cooperate to induce cell death during spermatogenesis^20^.

To summarize, human spermatozoa have the molecular machinery necessary for autophagy/mitophagy activation. Differences found in LC3-II/LC3-I ratio after incubation or by its pharmacological modulation with rapamycin and chloroquine might be indicating that autophagy is an active process in human spermatozoa. Autophagy activation may have a positive role in human sperm physiology, maintaining viability and motility. By contrast, when the normal autophagy/mitophagy flux is disrupted (observed by LC3 II, Tom20 and PINK1 accumulation by using chloroquine) a decrease in motility, intracellular ATP and calcium concentration was observed, together with a concomitant activation of caspases3/7, indicating a possible crosstalk between LC3 processing and proteins involved in the regulation of apoptosis. Finally, despite all the results are pointing out that a similar process of mitophagy observed in somatic cells is present and might be functional in human spermatozoa, future research works should be performed to reach a final conclusion. Also, we cannot discard that the autophagy-related processes described in the present report could be also involved in the regulation of additional functions in human spermatozoa, such acrosome reaction or capacitation.

## Methods

### Reagents

Chemical salts (NaCl, KCl, MgSO_4_, KPO_4_, 20 HEPES, sodium pyruvate, sodium lactate, CaCl_2,_ NaHCO_3_), formaldehyde, Triton X-100, deoxycholate, EGTA, EDTA, Na_3_VO_4_, glucose, Bovine Serum Albumin (BSA), Phosphate Buffered Saline (PBS), Chloroquine diphosphate salt (#C6628), anti-LC3B (#L7543), 2′,7′-Bis(2-carboxyethyl)-5(6)-carboxyfluorescein acetoxymethyl ester (#B8806) and anti-PINK1 (#HPA001931) were purchased from Sigma-Aldrich (St. Louis, MO). Ethidium Homodimer-1, Goat anti-Rabbit IgG (H+L) Secondary Antibody, Alexa Fluor 488/546 conjugate (#A-11034), CellEvent Caspase-3/7 Green Detection Reagent, TO-PRO-3 Iodide, MitoTracker Red CMXRos, MitoTracker Deep Red and LIVE/DEAD Sperm Viability Kit (#L7011) were purchased from Life Technologies (Eugene, OR). Complete EDTA-free, protease inhibitor cocktail was from Roche Diagnostics (Penzberg, Germany). Bradford reagent, Tris/Glycine/SDS buffer (10X) and Tris/Glycine buffer (10X) were from Bio-Rad (Richmond, CA). Anti-Beclin 1 pAb (#PD017Y), Anti-Atg16L pAb (#PM040Y), Anti-p62 (SQSTM1) pAb (#PM045Y), Anti-p62 (SQSTM1) (Human) mAb (#M162-3), Anti-Atg5 pAb (#PM050Y), Positive control for anti-LC3 (#PM036-PNY) were purchased from MBL International (Woburn, MA 01801). Anti-p-AMPKα1/2 (Thr 172) (#sc-33524), anti AMPKα1/2 (H-300) (#sc-25792) anti GAPDH (#sc-31915) and Tom20 (#sc-17764) antibodies were from Santa Cruz Biotechnology (Santa Cruz, CA). Anti-mTOR antibody (#2972) from Cell Signaling (Beverly, CA). Rapamycin (#553211) from MERCK (Billerica, MA). Anti-rabbit and anti-goat IgG horseradish peroxidase (HRP)-conjugated secondary antibodies and enhanced chemiluminescence detection reagents were from Pierce (Rockford, IL). InnovaCoat^®^ GOLD 10 nm Goat Anti-Rabbit IgG gold conjugate (10OD) (#218-0200) was from Innova Biosciences (Cambridge, UK). Hyperfilm ECL was from Amersham (Arlington Heights, IL), and nitrocellulose membrane was obtained from Schleicher & Schuell (Keene, NH).

### Ethics

This study was approved by the Institutional Review Board of the University of Extremadura and by the ethics committee of the Infantile Hospital (Badajoz, Spain) as well as in accordance with the Declaration of Helsinki. Written informed consent was obtained from all of the participants.

### Semen collection and preparation

Human semen was obtained from 33 healthy donors, in the age range of 25–40. Each subject was confirmed to be in good health by means of their medical histories and a clinical examination including routine laboratory test. The samples were collected by masturbation into sterile plastic jars, after 3 ± 5 days of sexual abstinence. After 1 h of incubation at 37 °C, routine semen analysis was performed. According to the World Health Organization criteria (2010) (progressive motility >32%, sperm cell concentration >15 × 10^6^ cells/ml; and sperm cells morphology >4%) only samples with the semen profile considered as normal were included in the study.

After semen liquefaction (1 hour at 37 °C), samples were centrifuged for 6 min at 250x g. the supernatant was discarded, and the pellet was resuspended in a medium containing: 120 mM NaCl, 15 mM NaHCO_3_, 4 mM KCl, 1.8 mM CaCl_2_, 1 mM MgCl_2_, 10 mM HEPES, 10 mM Na-Lactate, 5 mM glucose, 1 mM Na-piruvate. The pH of the solution was adjusted at 7.4.

To study autophagy in human spermatozoa, sperm cells (25 × 10^6^ cells/ml) were incubated for 2 hours at 37 °C in absence or presence of bafilomycin A1 (100 nM), chloroquine (50 μM) or rapamycin (500 nM). Bafilomycin A1, a known inhibitor of the late phase of autophagy, prevents maturation of autophagic vacuoles by inhibiting fusion between autophagosomes and lysosomes^44^. Chloroquine inhibits autophagy^68^ as it raises the lysosomal pH, which leads to inhibition of both fusion of autophagosome with lysosome and lysosomal protein degradation. Autophagy was activated with rapamycin which is an inhibitor of m-TOR, a negative regulator (in its active form) of autophagy^8^. Control samples were incubated in presence of the vehicle (DMSO) at the same concentration used for the treated samples.

### Western blotting

To separate the proteins according to their apparent molecular masses, SDS-PAGE was performed as previously described^70^. In brief, proteins were extracted and denatured by boiling for 10 min at 70 °C in a loading buffer supplemented with 5% mercaptoethanol. The protein content was calculated using the Bradford assay^73^. Fifteen micrograms of protein extract of spermatozoa was loaded and resolved by SDS PAGE on a 10% or 12% polyacrylamide gel. Immunoblotting was performed by incubating the membranes in blocking buffer overnight at 4 °C with primary antibodies (LC3, Atg5, Atg16, Beclin 1, p62, m-TOR, AMPKα 1/2, p-AMPKα 1/2, PINK1 and TOM20). Proteins from whole rat brain cells lysates were used as positive control for Atg5, Atg16, Beclin 1, p62, m-TOR, AMPKα ½, PINK1 and Tom20 proteins^74^,^75^,^76^. While commercial 293T cells with overexpressed human LC3 (10 μl) (#PM036-PNY) was used as positive control for LC3. Band intensity was quantified using the software ImageJ for Windows, version 1.50i.

### Indirect immunofluorescence and colocalization

Indirect immunofluorescence was performed as previously described^77^. After blocking, slides were incubated with primary antibodies (LC3, p62 and PINK1) overnight at 4 °C diluted in PBS containing 5% BSA (w/v). The following day, samples were extensively washed with PBS and further incubated for 45 min at RT with anti-rabbit IgG antibody conjugated with the Alexa 488/546 diluted to 1/500 in PBS containing 5% BSA (w/v). Finally, slides were thoroughly washed with PBS and examined in a Nikon Eclipse TE300 fluorescence microscope with a 100 X objective in oil immersion. Absence of nonspecific staining was assessed by processing the samples without primary antibody.

To study the colocalization of LC3 with mitochondria, sperm were incubated for 30 minutes with MitoTracker Red CMXRos (100 nM) at 37 °C. Then, samples were centrifuged and resuspended in PBS. Fifteen microliters of sample were spread on poly-L-lysine-coated slides and allowed to attach for 10 min. Then, protocol was followed as indicated above.

### Transmission electron microscopy analysis (TEM)

Cells were fixed in 2.5% of glutaraldehyde for 2 hours at RT. Cells were then washed twice with PBS at 37 °C and resuspended in 0.2 M sodium cacodylate buffer (pH 7.4) at 4 °C. Cells were dehydrated in graded ethanol series (30, 50, 70, 90 y 100%) and embedded in Epon 812. Ultrathing sections (Leica EM UC6) were mounted on cooper grids and stained for 10 min with 2% uranyl acetate and for 2 min with lead citrate. The sections were examined with a TEM 200 KV, Tecnai G220 (FEI, Oregon, USA) electron microscope.

### Immunogold labeling for LC3

Briefly, spermatozoa were fixed in 3% paraformaldehyde and 0.1% of glutaraldehyde for 1 hour at RT and washed in phosphate buffered saline (PBS) at 37 °C twice to remove excess fixative. Then, cells were resuspended in 0.2 M sodium cacodylate buffer (pH 7.4) at 4 °C. Further, cells were dehydrated in graded alcohol, infiltrated in LR White resin, polymerized in a vacuum oven at 45 °C for 48 hr and 60 nm ultrathin sections were cut and placed on cooper grids for post-embedding immunogold labeling for anti-LC3 Ab. Potential non specific labeling was blocked by incubating the sections in PBS containing 1% of bovine serum albumin at room temperature for 1 hr. Sections were then incubated overnight at 4 °C with anti-LC3 dilution of 1:100 in PBS + 0.1% of BSA buffer. Incubated in 10 nm colloidal gold conjugated goat anti-rabbit IgG secondary Ab at 1:100 dilution for 3 hours at RT. The sections were then subsequently washed in PBS and counterstained in 2% of uranyl acetate and 0.2% of lead citrate. To assess the specificity of the immunolabeling, a negative control was carried out in those sections of sperm that were labeled with colloidal gold conjugated secondary Ab without the primary Ab.

### Flow cytometry analysis

Flow cytometric analyses were conducted using a MACSquant Analyzer 10 (Miltenyi Biotech) flow cytometer equipped with three lasers emitting at 405, 488 and 635 nm and 10 photomultiplier tubes (PTMs). Dyes were detected in V1 (excitation (Ex) 405 emission (Em) 450/50), B1 (Ex 488 filter 525/50 and B3 (Ex 488 filter 655–730 (655LP+ split 730)). The system was controlled with the MACSquantify software. Forward and sideways light scatter were recorded for a total of 30,000 events per sample. Non-sperm events were eliminated by gating the DNA-containing sperm population after Hoechst 33342 (0.5 μM) staining for 30 min at 37 °C.

### Assessment of human spermatozoa viability

Viability assessment was performed using the LIVE/DEAD Sperm Viability Kit. In brief, 100 μl (25 × 10^6^ cells/ml) of sample was resuspended in 900 μl of phosphate buffered saline and was incubated at room temperature for 10 min with SYBR 14 (100 nM) in the dark. Further, samples were stained with propidium iodide (1 μM) and incubated for 5 additional minutes before analysis in the flow cytometer.

### Detection of active Caspase 3 and 7

Detection of active Caspase 3 and 7 was carried out using “CellEvent Caspase-3/7 Green Detection Reagent” which is a four amino acid peptide (DEVD), conjugated to a nucleic acid binding dye. After activation of caspase-3/7 in apoptotic cells, the DEVD peptide is cleaved enabling the dye to bind to DNA and produce a bright, fluorogenic response. For this purpose, 100 μl (25 × 10^6^ cells/ml) of sample was resuspended in 900 μl of phosphate buffered saline and was incubated for 30 minutes at 37 °C with Caspase-3/7 reagent (2 μM) and Hoechst 33342 (0.5 μM). Further, samples were stained with ethidium homodimer (1 μM) and incubated for 5 additional minutes before analysis in the flow cytometer.

### Intracellular pH measurement

The intracellular pH (pHi) of human sperm was measured by fluorescent pH-indicator 2,7-bicarboxyethy l–5, 6-carboxyfluorescein- acetoxymethylester (BCECF-AM) which is a neutral lipophilic form of bis carboxyfluorescein which diffuses freely through the plasma membrane. In the cell, it is hydrolyzed by esterases, releasing the BCECF which is retained within the cytoplasm. The fluorescence intensity of BCECF is dependent upon the pH. After sperm incubation for 2 hours with or without chloroquine (50 μM) or rapamycin (500 nM), sperm were centrifuged and the pellet was resuspended in 1 ml of phosphate-buffered saline (PBS, pH = 7.4). Sperm (1 × 10^6^/ml) were then loaded with 100 nM BCECF-AM (final concentration) and incubated (37 °C, 30 min) in dark. Following incubation, sperm were centrifuged, washed and resuspended in 1 ml of PBS. Dead cells were detected with TO-PRO-3 Iodide (1/10,000). For the intracellular pH calibration curve sperm were loaded with BCECF. Cells were centrifuged and the pellet was resuspended in 1 ml of PBS at various extracellular pHs (6, 6.5, 7.0, 7.5, 8) and TO-PRO-3 Iodide was added. Then sperm were treated with 1 μM Triton-X100. pHi was calculated following the manufacturer’s protocol.

### MitoTracker Deep Red determination

For this purpose, 100 μl (25 × 10^6^ cells/ml) of sample was resuspended in 900 μl of phosphate buffered saline and was incubated for 30 minutes at 37 °C with MitoTracker Deep Red reagent (100 nM) and Hoechst 33342 (0.5 μM). Further, samples were stained with ethidium homodimer (1 μM) and incubated for 5 additional minutes before analysis in the flow cytometer. Mean fluorescence in the viable cell population was plotted and normalized against that of untreated cells.

### Measurement of intracellular free calcium concentration ([Ca^2+^]c)

After 90 minutes of incubation with the different drugs, cells were loaded with fura-2 by incubation with 4 μM fura-2 acetoxymethyl ester (Fura-2 AM). The incubation continued for a further 30 minutes at room temperature, according to a procedure published elsewhere^78^. Then, cells were washed and used within the next 2–4 hours. Fluorescence was recorded from 2 mL aliquots of magnetically stirred cellular suspension (2 × 10^8^ cells/mL) at 37 °C using a Shimadzu spectrofluorophotometer with excitation wavelengths of 340 and 380 nm and emission at 505 nm. Changes in [Ca^2+^]c were monitored by using the fura-2 340/380 nm fluorescence ratio and were calibrated according to the method of Grynkiewicz *et al*.^79^.

Calcium entry and release were estimated using the integral of the rise in [Ca^2+^]c for 2.5 min after addition of progesterone. Both calcium entry and release are expressed as nanomolar taking a sample every second (nM·s), as previously described^80^.

### ATP determination

ATP concentration was measured with ATP Determination Kit from Molecular probes (A22066). After 2 h of incubation, 200 μl of samples (30 × 10^6^ cells/ml) were directly frozen in liquid nitrogen and kept at −80 °C. Samples were thawed in ice and centrifuged for 15 min at 10.000 g at 4 °C. Supernatant was then recovered and maintained in ice until ATP measurement^81^. Reactions were prepared following manufacturer’s instructions. Measurement of the signal produced was performed in Tecan Infinite M200 plate reader. ATP concentration was calculated through a standard curve and normalized with the total protein concentration in each sample.

### Sperm motility

Sperm kinematics was assessed in fresh and incubated samples using a computer-assisted semen analysis (CASA) system (ISAS 1.0.6; Proiser S.L., Valencia, Spain). 2 μl of semen was loaded in 20-μm-deep Leja (Amsterdam, the Netherlands) chambers placed in a thermostatized microscope stage (37 °C). The analysis was based on the examination of 25 consecutive, digitalized images obtained from a single field using x10 negative phase contrast objective. Images were taken with a time lapse of 1 sec; therefore, the image capture speed was one every 40 ms. Number of objects incorrectly identified as spermatozoa was manually minimized on the monitor using the playback function. With respect to the setting parameters for the program, spermatozoa with a velocity <10 μm/s were considered immotile; spermatozoa with a velocity >35 μm/s were considered rapid spermatozoa.

### Statistical analysis

Data were first examined using the Kolmogorov–Smirnov test to determine their distribution. Multivariate analysis of variance was performed (ANOVA) followed by post hoc Tukey’s test was used in all studies. Pearson correlation test was used to study the correlations between the semen parameters in fresh samples and LC3-II/LC3-I ratio. Significance was set at p < 0.05. All analyses were performed using SPSS version 17.0 for Windows (SPSS Inc., Chicago, IL, USA).

## Additional Information

**How to cite this article**: Aparicio, I. M. *et al*. Autophagy-related proteins are functionally active in human spermatozoa and may be involved in the regulation of cell survival and motility. *Sci. Rep.* **6**, 33647; doi: 10.1038/srep33647 (2016).

## Figures and Tables

**Table 1:** Correlation between LC3-II/I ratio and semen parameters.

Parameters	LC3-II/I ratio	
	Correlation	Significance
Ejaculate volume	−0.061	p = 0.787
pH	0.008	p = 0.972
Concentration (spz/ml)	−0.482	p = 0.023*
Concentration (spz/ejaculate)	−0.508	p = 0.016*
Total motility	−0.262	p = 0.264
Progressive motility	−0.446	p = 0.042*
Round cells (cells/ml)	0.139	p = 0.536
% Normal cells	−0.182	p = 0.430
Viability	0.458	p = 0.032*

Correlation was performed with semen parameters collected from 33 subjects and values of LC3II/LC3I ratio, obtained as commented in Fig. 2. Within a raw asterisk means significant correlation between LC3-II/I ratio and the parameter under study.

**Table 2:** Effect of chloroquine and rapamycin in human sperm velocity parameters.

Velocity Parameters	Control	Chloroquine (50 μM)	Rapamycin (500 nM)
VCL (μm/s)	64.24 ± 4.54	60.90 ± 6.28	66.52 ± 5.44
VSL (μm/s)	25.35 ± 2.09	18.53 ± 1.90*	28.25 ± 1.15
VAP (μm/s)	36.52 ± 2.30	31.88 ± 2.78	38.84 ± 2.60
LIN (%)	39.76 ± 2.18	30.60 ± 1.56	43.28 ± 3.10
STR (%)	69.37 ± 2.44	57.95 ± 2.86*	73.36 ± 3.10
WOB (%)	57.18 ± 1.37	52.78 ± 1.33*	58.78 ± 1.99
ALH (μm)	3.06 ± 0.13	3.09 ± 0.47	2.85 ± 0.08
BCF (Hz)	9.34 ± 0.62	6.49 ± 1.36	9.44 ± 0.58

Velocity parameters were assessed by CASA system after 2 hours of incubation with chloroquine (50 μM) or rapamycin (500 nM). VCL: Curvilinear velocity. VSL: Linear velocity. VAP: Mean velocity. LIN: Linearity coefficient. STR: Straightness coefficient. WOB: Wobble coefficient. ALH: Mean lateral head displacement. BCF: Frequency of head displacement. Values represent the mean + SEM of four separate experiments (*p < 0.05 compared with untreated control containing only vehicle).
